# Nurses’ roles in changing practice through implementing best practices: A systematic review

**DOI:** 10.4102/hsag.v27i0.1776

**Published:** 2022-05-25

**Authors:** Wilma ten Ham-Baloyi

**Affiliations:** 1 Faculty of Health Sciences, Nelson Mandela University, Port Elizabeth, South Africa

**Keywords:** best practices, changing practice, implementation, nurses, roles, systematic review

## Abstract

Nurses play an important role in the implementation of best practices. However, the role of nurses in changing practice by implementing best practices requires further exploration. No systematic review was found that summarised the best available evidence on the roles of nurses in changing practice through the implementation of best practices. This study summarised the best available evidence on the roles of nurses in changing practice through the implementation of best practices. A systematic review was used to search for studies in the English language, where a best practice was implemented in a clinical context and which included findings regarding the roles of nurses when implementing best practices. Scopus, EBSCOhost (Academic Search Ultimate, APA PsycInfo, CINAHL with Full Text, ERIC, Health Source: Nursing/Academic Edition, MasterFILE Premier, MEDLINE Complete), PUBMED, and ScienceDirect databases were searched from January 2013 to June 2021. The search generated 1343 citations. After removing duplicates and applying eligibility criteria, 27 studies were included. Five definite roles were identified as follows: leadership, education and training, collaboration, communication and feedback and development and tailoring of the best practice. These roles are interrelated, but equally crucial in order to implement best practices. This study found five interrelated but equally crucial nurse roles in changing practice through the implementation of best practices.

**Contribution:**

The study’s findings and gaps identified can be used for further nursing research, improving practice change and health outcomes through the implementation of best practices and the role nurses can play in this process.

## Introduction

Globally, in the last decades, there have been rapid changes in healthcare and nursing practice, based on the best available evidence, to improve patient, nursing and organisational outcomes whilst, at the same time, using resources efficiently (Cullen & Donahue 2016; Salmond & Echevarria 2017). A sustained change in practice through the implementation of best practices is required to improve health and patient outcomes such as length and costs of hospital stay (Leviton & Melichar 2016).

Research findings based on rigorous methods that have been identified as best evidence and evidence-based products such as evidence-based innovations, interventions, strategies, practice improvements, guidelines, initiatives, programmes or recommendations (in this study referred to as ‘best practices’) assist in changing health and nursing practice (International Council of Nurses 2012). However, implementation of best practices remains problematic (Greenhalgh, Howick & Maskrey 2014). Innovative ways are required to firstly translate best evidence, which is the application of knowledge (Graham et al. 2018) and thereafter implement the best practice. This is especially relevant for a healthcare and nursing environment that is increasingly competitive and has to operate in a cost-effective way (Salmond & Echevarria 2017).

Furthermore, there are various stakeholders who influence implementation of best practices or change in practice and these stakeholders are also affected by change in practice (Agency for Healthcare Research and Quality [AHRQ] 2016). Thus, there is a strong drive for stakeholders to be actively engaged in and to make committed decisions about changing practice (Norris et al. 2017). To do so, the roles of the various stakeholders in changing practice – which includes patients and their families, the nurses and other healthcare practitioners and the managers at micro, meso and macro levels of the health system – need to be understood. Understanding the roles of these stakeholders in changing practice will assist in a more effective and efficient implementation and uptake of innovative best practices and, ultimately, will improve healthcare outcomes (Leviton & Melichar 2016).

Nurses, as one of the stakeholders, play an important role in the implementation of best practices. However, the role of nurses in changing practice by implementing best practices is not always well understood (Kristensen, Nymann & Konradsen 2016). No systematic review was found that summarised the best available evidence on the roles of nurses in changing practice through the implementation of best practices. This review therefore aimed to summarise the best available evidence on the roles of nurses in changing practice through the implementation of best practices.

## Methods

### Design

A systematic review was conducted to collect data, identify high-quality relevant studies and to synthesise the findings in a rigorous and comprehensive way so that a comprehensive picture of current best available evidence could be provided. In this case, the best available evidence on the roles of nurses in changing practice through the implementation of best practices as a preliminary search did not yield any systematic reviews. The systematic review was conducted according to the Systematic Review guidelines of the Joanna Briggs Institute (JBI). The following review question was formulated: ‘What is/are the role(s) of nurses in changing practice when implementing best practices’?

### Search methods

#### Sources of evidence

The following databases were searched: Scopus, EBSCOhost (Academic Search Ultimate, APA PsycInfo, CINAHL with Full Text, ERIC, Health Source: Nursing/Academic Edition, MasterFILE Premier, MEDLINE Complete), Pubmed and ScienceDirect.

#### Keywords

A broad combination of keywords was used to search the literature on the topic. A set of keywords per database was selected to yield the most relevant studies. The following keywords were used: role OR function AND nurse OR nurses OR nursing AND implement* AND best practice OR best practices.

#### Inclusion criteria and exclusion criteria

Studies of the following levels of evidence, according to JBI (2016), were included: Level I Experimental studies: (c) randomised controlled trials (RCT), (d) pseudo-RCTs; Level II Quasi-experimental studies: (c) quasi-experimental prospectively controlled study, (d) pre-test, post-test/retrospective control group; Level III Observational Analytical studies: (c) cohort study with control group, (d) case controlled study, (e) observational study without a control group; Level IV Observational Descriptive studies: (b) cross-sectional study, (c) case series, (d) case studies. Only those studies published in English from January 2013 to June 2021 were eligible for selection.

Studies were included where a best practice was implemented in a healthcare or clinical context (inside or outside a hospital setting where nursing care is rendered, e.g. old age setting), published in English, which included findings regarding the roles of nurses when implementing best practices. Systematic types of reviews and non-research studies were excluded as well as studies that were not implementing best practices (e.g. studies where no intervention was implemented or not described, studies regarding the views on the role of nurses implementing best practices in general or general perceived facilitators and barriers).

#### Librarian

The entire search strategy, including the choice of keywords and electronic databases was conducted with the assistance of an experienced librarian from the Nelson Mandela University. Similar assistance was provided in obtaining studies, some via Inter-Library Loan services.

### Search outcome

For this study, the following steps for selection were followed:

1. The researcher read titles and abstracts (whereby irrelevant studies were excluded according to the pre-determined inclusion and/or exclusion criteria).
2. Possible relevant literature was selected in order to obtain full-text. The researcher read the full text of potentially relevant studies and selections for inclusion were made according to pre-determined inclusion and/or exclusion criteria.
3. When no full text could be obtained to determine inclusion and/or exclusion of an article, Inter-Library Loan services was used and authors were contacted.

**FIGURE 1:**
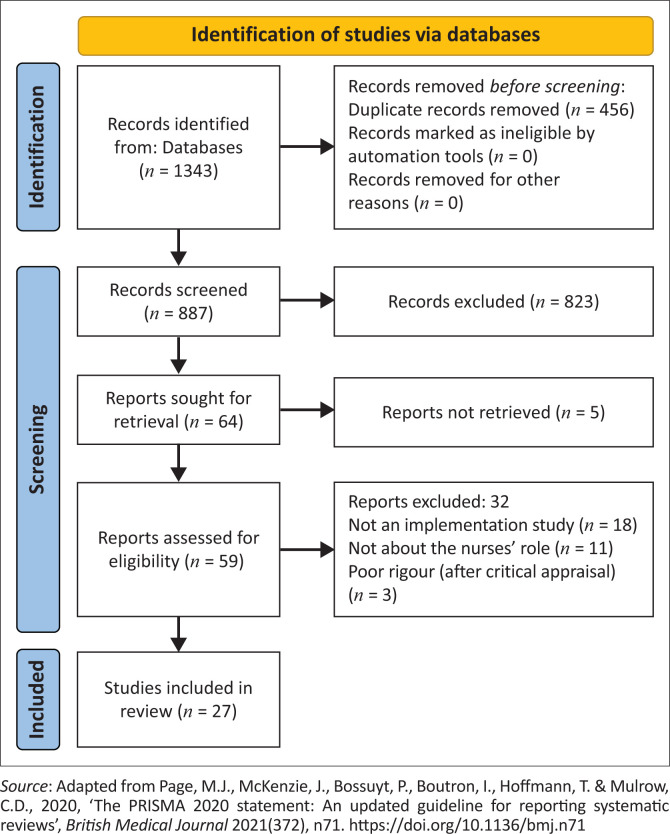
Search and selection process.

EndNote X9 was used for data management, obtaining full-texts and for deduplication. The search and selection process is outlined in Figure 1.

As a result of the literature search, 1343 initial hits were imported from electronic databases. After removing 456 duplicates, 887 titles and abstracts were read. A total of 823 were excluded as they did not meet inclusion criteria. From the remaining titles, total of 59 full-texts were obtained as five articles could not be located. Reading of the 59 articles led to exclusion of a further 29 articles, based on the study criteria.

### Critical appraisal

A total of 30 studies fulfilled the review criteria and were included for critical appraisal. Appraisal was done using various tools, according to the different research designs or levels of evidence of the literature, including the various 64 JBI (Pearson, Jordan & Munn 2012) tools, including: checklist for analytical cross-sectional studies (*n* = 2), checklist for cohort studies (*n* = 1), checklist for qualitative research (*n* = 7); checklist for quasi-experimental studies (*n* = 2) (JBI 2021).

The following critical appraisal tools were found most suitable but were not available through JBI: The Strengthening the Reporting of Observational Studies in Epidemiology (STROBE) Statement: guidelines for reporting observational studies (Von Elm et al. 2007) (*n* = 16), Mixed Methods Appraisal Tool (MMAT) (Hong et al. 2018) (*n* = 1) and the Effective Practice and Organization of Care Risk of Bias (EPOC RoB) tool for retrospective observational studies such as audits, developed by Cochrane (eds. Higgins et al. 2019) (*n* = 1).

To reduce bias in review selection and to ensure that the appraisal was performed in a rigorous way, whilst allowing for appropriate consensus, the appraisal was conducted by two reviewers independently using the same critical appraisal tools. The outcome of the critical appraisals was shared amongst the researcher and independent reviewer during a meeting and consensus was achieved in terms of inclusion or exclusion of literature. Out of the total of 30 articles that were included for critical appraisal, three observational studies using audits were excluded because of weak rigour (see Figure 1).

### Data extraction

Data extraction from the sample was done by recording relevant elements of studies regarding the topic in a tabular format. Headings in the table included: study reference, design, level of evidence, sample and setting, best practice and change strategy and findings relevant to the topic.

### Data synthesis

For this review because of the heterogeneous nature of the study designs included thematic analysis, which was done to synthesise the extracted findings of each study, followed by a classification of findings and a summary of findings under thematic headings (as formulated in Academy of Nutrition and Dietetics [2012]).

### Ethical considerations

This study obtained ethical approval from the University’s Faculty Postgraduate Studies Committee (ethics number: H19-HEA-NUR-008). The author adhered to the principles of honesty and transparency in reporting the data. In line with recommendations of Vergnes et al. (2010), participant consent was not obtained because this study had no participants.

## Results

### Quality of evidence

The majority of studies (*n* = 17) were observational analytical studies: Level III(e) evidence and Level IV evidence (*n* = 7, of which *n* = 4 IV(b) and *n* = 3 IV(d)). Two other studies (*n* = 2) included Level II(d) evidence. One (*n* = 1) mixed method study included both Level III(e) and Level IV(b) evidence (JBI 2016).

### Healthcare or clinical context

Studies were from a variety of healthcare or clinical contexts, with the majority (*n* = 20) from a hospital setting. Of these, *n* = 14 were conducted in specialised hospital-based settings, including: medical and surgical wards (*n* = 2) (Siegel 2020; Travers et al. 2018), paediatric settings (*n* = 2) (Rosenberg et al. 2016; Yu et al. 2017), postnatal ward (*n* = 1) (Anderson & Kynoch 2017), neonatal intensive care unit (*n* = 1) (Ceballos et al. 2013), surgical ward (*n* = 1) (Hu et al. 2019), haemodialysis centre (*n* = 1) (Jia et al. 2016), haematology–oncology (*n* = 1) (Naseer et al. 2017), orthopaedic ward (*n* = 1) (Ong et al. 2017), medical ward (*n* = 1) (Ullrich, McCutcheon & Parker 2015), intensive care unit (*n* = 1) (Chiwaula et al. 2021), in-patient rehabilitation (*n* = 1) (Mullins 2021) and a neurology department (Sheng et al. 2020).

A total of five (*n* = 5) studies were from outside hospital settings, including long-term care (*n* = 2) (Kilpatrick et al. 2020, Mitchell 2017), homecare centres (*n* = 1) (Bayly et al. 2018), acute ambulatory settings (*n* = 1) (Chong et al. 2013) and a general practitioner (GP) practice (*n* = 1) (Williams et al. 2020).

Two (*n* = 2) studies were conducted inside and outside hospital settings. One of these studies was conducted in both a residential age-care facility and hospital setting (Ullrich, McCutcheon & Parker 2014) and the other study was conducted in a hospital setting (inpatient, acute care medical or surgical, intensive care units) and in a long-term care setting (progressive care/stepdown, community home, long-term care, rehabilitation, palliative/hospice care and spinal cord injury) (Becker et al. 2020).

### Country

Studies were conducted in a variety of countries, including Australia (*n* = 6), United States of America (*n* = 6), Canada (*n* = 4), China (*n* = 4), Singapore (*n* = 3), United Kingdom (*n* = 2), Malawi (*n* = 1) and Thailand (*n* = 1).

### Best practices and implementation strategies for change

In total, seven (*n* = 7) best practices and 11 (*n* = 11) implementation strategies for change were identified from the included studies. The best practices included: best practice, intervention, strategy, guideline, initiative, programme and recommendation. The implementation strategies included: educational sessions or workshops, (development of) educational material, champion or knowledge broker, discussions, evaluation and feedback, development of an evidence-based practice (EBP) product, employing team or specialists, meetings, observations, equipment, assessments or examinations. Table 1 outlines the best practice and implementation strategies for change, per included study.

**TABLE 1:** Best practices and implementation strategies for change (*n* = 27).

References	Best practices							Implementation strategies											
	Best practice	Intervention	Strategy	Guideline	Initiative	Program	Recommendation	Educational sessions/workshops	(Development of) educational material	Champion/knowledge broker	Discussions	Evaluation and feedback	Development of EBP product	Employing team/specialists	Meetings	Observations	Equipment	Assessments/ examinations	Total number of implementation strategies per study
Allen et al. (2018)	x	-	-	-	-	-	-	x	-	x	-	-	-	-	x	-	-	-	*n* = 3
Anderson and Kynoch (2017)	x	-	-	-	-	-	-	x	x	x	x	-	-	-	-	-	-	-	*n* = 4
Bayly et al. (2018)	-	-	x	-	-	-	-	-	-	x	-	-	-	-	x	-	-	-	*n* = 2
Becker et al. (2020)	-	-	-	-	-	-	x	-	x	x	-	x	x	-	x	-	-	-	*n* = 5
Ceballos et al. (2013)	-	x	-	-	-	-	-	-	x	x	-	x	-	-	-	-	-	-	*n* = 3
Chiwaula et al. (2021)	-	x	-	-	-	-	-	x	-	-	-	x	x	-	-	-	-	-	*n* = 3
Chong et al. (2013)	x	-	-	-	-	-	-	x	-	-	x	-	-	-	-	x	-	-	*n* = 3
Fleiszer et al. (2015)	-	-	-	x	-	-	-	x	-	x	-	x	-	x	-	-	x	-	*n* = 5
Fleiszer et al. (2016)	-	-	-	x	-	-	-	x	-	x	-	x	-	x	-	-	-	-	*n* = 4
Hu et al. (2019)	-	-	x	-	-	-	-	-	x	-	x	x	-	-	-	-	-	-	*n* = 3
Jia et al. (2016)	x	-	-	-	-	-	-	x	x	-	-	x	x	-	-	-	x	x	*n* = 6
Kilpatrick et al. (2020)	-	x	-	-	-	-	-	x	-	-	-	-	x	x	-	-	-	-	*n* = 3
Mitchell (2017)	-	-	x	-	-	-	-	x	-	-	-	-	-	x	-	-	-	-	*n* = 2
Monkong et al. (2020)	x	-	-	-	-	-	-	-	-	x	x	-	-	-	-	x	-	-	*n* = 3
Mullins (2021)	-	-	-	x	-	-	-	-	x	-	-	-	-	x	-	x	-	-	*n* = 3
Naseer et al. (2017)	x	-	-	-	-	-	-	x	-	-	x	-	-	-	-	-	-	-	*n* = 2
Ong et al. (2017)	x	-	-	-	-	-	-	x	x	-	x	-	x	-	-	-	-	-	*n* = 4
Rosenberg et al. (2016)	x	-	-	-	-	-	-	x	-	x	-	x	-	x	-	-	-	-	*n* = 4
Shade et al. (2020)	-	x	-	-	-	-	-	x	-	-	-	-	-	x	x	-	-	-	*n* = 3
Sheng et al. (2020)	-	-	x	-	-	-	-	x	x	-	-	-	x	-	-	-	-	x	*n* = 4
Siegel (2020)	-	-	-	x	-	-	-	x	x	-	-	-	-	-	-	-	-	-	*n* = 2
Travers et al. (2018)	-	-	-	-	x	-	-	x	-	x	-	-	-	-	-	-	-	-	*n* = 2
Ullrich et al. (2014)	x	-	-	-	-	-	-	-	-	-	-	-	-	-	x	x	-	-	*n* = 2
Ullrich et al. (2015)	x	-	-	-	-	-	-	-	x	-	-	-	-	-	-	x	-	-	*n* = 2
Williams et al. (2019)	-	-	-	-	-	x	-	-	x	-	x	-	-	-	x	-	-	-	*n* = 3
Williams et al. (2020)	-	x	-	-	-	-	-	-	-	-	-	-	-	-	x	-	-	-	*n* = 1
Yu et al. (2017)	-	x	-	-	-	-	-	-	x	-	x	-	x	-	-	-	x	-	*n* = 4

**Total**	***n* = 10**	***n* = 6**	***n* = 4**	***n* = 4**	***n* = 1**	***n* = 1**	***n* = 1**	***n* = 16**	***n* = 12**	***n* = 10**	***n* = 8**	***n* = 8**	***n* = 7**	***n* = 7**	***n* = 7**	***n* = 5**	***n* = 3**	***n* = 2**	**-**

EBP, evidence-based practice.

As outlined in Table 1, included studies indicated a variety of implemented best practices, with best practice or intervention being mostly identified as best practice. Various implementation strategies for change were used, but most studies used more than one strategy, up to six strategies and had an element of education and leadership.

## Roles of nurses

Eleven (*n* = 11) of the included studies were nurse-led quality improvement projects, in which a team was formed in the clinical setting with nurses who took the lead and facilitated change through the implementation of the best practice in this setting (Anderson & Kynoch 2017; Bayly et al. 2018; Ceballos et al. 2013; Chong et al. 2013; Hu et al. 2019; Jia et al. 2016; Monkong et al. 2020; Naseer et al. 2017; Ong et al. 2017; Travers et al. 2018; Yu et al. 2017).

Five definite roles were identified: leadership, education and training, collaboration, communication and feedback and development and tailoring of the best practice. These roles are further described in the following subsections.

### Leadership

Leadership played a role in almost all studies (*n* = 21). This could be individuals, for example, a clinical champion (Allen et al. 2018; Becker et al. 2020), a (clinical) team leader (Anderson & Kynoch 2017; Chong et al. 2013), a project leader (Hu et al. 2019; Mullins 2021; Yu et al. 2017) or nurse leader (Ceballos et al. 2013), a nurse clinician (Nazeer et al. 2017; Ong et al. 2017), a knowledge broker (Bayly et al. 2018), a practice facilitator (Shade et al. 2020), Facilitator CogChamps (Travers et al. 2018), a programme or project coordinator (Fleiszer et al. 2015, 2016; Monkong et al. 2020) or an audit team leader (Jia et al. 2016). In some studies, the leader was the researcher (Mitchell 2017) or part of the research team (Kilpatrick et al. 2020; Rosenberg et al. 2016; Williams et al. 2019).

Roles of leaders included:

- recruitment of participants (Becker et al. 2020)
- facilitating the implementation of the best practice (Anderson & Kynoch 2017)
- creating educational material (e.g. a computer-based educational module, completion of a comprehensive literature review to inform the educational intervention) (Ceballos et al. 2013; Yu et al. 2017).
- communication (e.g. sending staff electronic communication with information about the best practice and why practice changes were necessary [Ceballos et al. 2013]; explain roles and responsibilities to every team member in fortnightly meetings [Chong et al. 2013]; introduce the project to the members and project timelines [Becker et al. 2020; Naseer et al. 2017])
- data analysis, interpretation of data and report writing (Ceballos et al. 2013; Chong et al. 2013; Yu et al. 2017)
- managing the project, process control and promotion and keeping timelines (Monkong et al. 2020; Mullins 2021; Yu et al. 2017)
- role modelling in terms of enthusiasm (Chong et al. 2013; Yu et al. 2017; Williams et al. 2019), commitment (Chong et al. 2013; Williams et al. 2019), approachability, sound clinical knowledge and legitimacy (Williams et al. 2019), ability to communicate clearly, being tenacious (keep on going when some nurses showed disinterest) and being able to think creatively about patients and patient care (Travers et al. 2018)

### Education and training

Education and training were found to play a big role in nurses implementing best practices in the majority of the studies (*n* = 21). Education and training were sometimes provided by the nurse leader (Shade et al. 2020; Travers et al. 2018; Yu et al. 2017).

Education focused mainly on nursing/healthcare staff in terms of educational sessions (Mitchell 2017; Monkong et al. 2020; Naseer et al. 2017), such as ward-based in-service training (Anderson & Kynoch 2017; Chong et al. 2013; Hu et al. 2019), (1-day) training/workshop (Chiwaula et al. 2021; Fleiszer et al. 2015; Shade et al. 2020; Travers et al. 2018), two half-day training sessions including formal presentations, video demonstration of the delivery of the best practice, participative learning and practice sessions (Williams et al. 2020), an educational programme (Yu et al. 2017), a lecture (Siegel 2020), a multimedia educational framework (Rosenberg et al. 2016; Sheng et al. 2020), online educational videos (Siegel 2020), online modules or courses (Bayly et al. 2018; Ceballos et al. 2013; Williams et al. 2019), along with educational tools such as notebooks containing hard copies of online training (Ceballos et al. 2013).

Other educational tools and strategies included: user guide (Kilpatrick et al. 2020), demonstration of sample scripts (Ong et al. 2017), scripts to educate patients (Siegel 2020) and documents and ‘informants’ with knowledge (Fleiszer et al. 2015). Training of the stakeholders (e.g. volunteer practice change advocates) in the implementation of best practices (Fleiszer et al. 2015) and daily practice under supervision (Chong et al. 2013) was also done.

As part of the implementation, nurses also used patient education through the development and use of educational tools such as hand-outs (Anderson & Kynoch 2017), a patient education leaflet (Hu et al. 2019), an educational booklet (Bayly et al. 2018) and pamphlets, posters or slides using an iPad (Jia et al. 2016).

The impact of education and training as part of the implementation of best practices for nurses was that it imparted knowledge, increased nurses’ empathetic and adaptable problem-solving skills, raised awareness and compliance with best practices amongst nurses and made nurses more confident in their roles (Allen et al. 2018; Naseer et al. 2017; Shade et al. 2020; Travers et al. 2018; Williams et al. 2019; Yu et al. 2017).

### Collaboration

Changing practice was often performed through a collaborative effort, as found in most studies (*n* = 20). For example, the nurse often led and formed a team with other nurses (Chiwaula et al. 2021; Chong et al. 2013; Fleiszer et al. 2016; Jia et al. 2016; Mitchell 2017; Naseer et al. 2017; Ong et al. 2017; Ullrich et al. 2015; Yu et al. 2017). Alternatively, a nurse led and collaborated with multiple health professionals besides nurses (specialists and managers) in a team in order to implement the best practice (Allan et al. 2018). Such teams including mainly medical staff/directors (Ceballos et al. 2013; Hu et al. 2019; Kilpatrick et al. 2020; Monkong et al. 2020; Rosenberg et al. 2016; Shade et al. 2020), as well as other professions such as a lactation consultant (Anderson & Kynoch 2017), a researcher (Bayly et al. 2018), a clinical pharmacist (Rosenberg et al. 2016), a respiratory specialist (Ceballos et al. 2013) and a dietician (Mullins 2021). One study also collaborated with a patient’s family as part of the interventions (Mullins 2021).

The various team members or stakeholders served as support (Anderson & Kynoch 2017; Chong et al. 2013; Kilpatrick et al. 2020; Naseer et al. 2017; Travers et al. 2018). Collaboration overcame challenges (Chong et al. 2013), enhanced care policies based on best evidence (Rosenberg et al. 2016), enhanced accountability (Fleiszer et al. 2016), raised collective awareness and expectations for practice, leading to a change in culture, empowerment, mutual respect and communication (Ceballos et al. 2013).

### Communication and feedback

Besides education, communication and feedback by nurses played an important role in the implementation of the best practice and often facilitated the implementation and uptake of the best practice, as found by more than half (*n* = 16) of the studies. Pre-implementation of the best practice, communication was done through meetings or brain storming sessions with ward stakeholders to discuss current practices (Monkong et al. 2020) or outlining the project audit (data collection) and timelines (Anderson & Kynoch 2017; Hu et al. 2019).

During the implementation, discussions or (feedback) meetings were held to present baseline audits and to gather feedback about the project (Anderson & Kynoch 2017; Becker et al. 2020; Chong et al. 2013; Fleiszer et al. 2015; Hu et al. 2019; Mullins 2021; Naseer et al. 2017; Shade et al. 2020), to discuss barriers to the implementation of the best practice (Jia et al. 2016; Mullins 2021; Naseer et al. 2017; Ong et al. 2017; Shade et al. 2020; Ullrich et al. 2014, 2015; Yu et al. 2017) and how to overcome the barriers (Mullins 2021; Shade et al. 2020; Ullrich et al. 2014, 2015; Yu et al. 2017), to develop and further improve strategies for implementation (Ceballos et al. 2013; Naseer et al. 2017; Ong et al. 2017) and to discuss progress (Rosenberg et al. 2016).

Post-implementation communication was used to brief stakeholders regarding the evaluation of the intervention (Chong et al. 2013; Fleiszer et al. 2015; Ong et al. 2017; Ullrich et al. 2015), to discuss how to overcome future barriers (Ong et al. 2017; Shade et al. 2020) or to celebrate success (Shade et al. 2020). Communication was also done online regarding the intervention (Becker et al. 2020; Ceballos et al. 2013), using emails (Naseer et al. 2017; Rosenberg et al. 2016) and text messages (Naseer et al. 2017).

Ongoing communication and feedback assisted in facilitating the implementation of best practices as it led to the creation of a supportive rapport, which increased engagement (Anderson & Kynoch 2017), compliance (Hu et al. 2019) and both technical and personal support for the project (Anderson & Kynoch 2017; Hu et al. 2019). It further helped to keep the knowledge translation strategies on track (Bayly et al. 2018; Shade et al. 2020), enhance the collaborative processes, enhance the ability to learn from peers’ professional experiences and share and use new information learned (Bayly et al. 2018). Finally, ongoing communication helped to identify barriers (Ceballos et al. 2013; Hu et al. 2019) and enhanced sustainability of the change (Becker et al. 2020).

### Development and tailoring of the best practice

Nurses play a role in the development and tailoring of the best practice, including the development of intervention materials as part of the implementation, as found in more than half (*n* = 16) of the included studies. The roles of nurses mainly involved developing an action plan (knowledge translation) or strategies, which was often done through informal discussions with nursing/midwifery staff and identifying barriers and facilitators of planned practice change (Anderson & Kynoch 2017; Bayly et al. 2018; Becker et al. 2020; Chong et al. 2013; Hu et al. 2019; Jia et al. 2016; Monkong et al. 2020; Naseer et al. 2018; Ong et al. 2017). Development of the best practice activities were also done (Sheng et al. 2020; Ullrich et al. 2015).

Other roles included developing educational material based on best evidence as part of the best practice, such as educational content, posters and hand-outs (Anderson & Kynoch 2017; Travers et al. 2018), videos and slides and a nursing newsletter (Becker et al. 2020), a computer-based educational module (Ceballos et al. 2013) and notebooks containing hardcopies of the online training information or information/resource booklet (Bayly et al. 2018; Ceballos et al. 2013).

Checklists to assist nurses to care for patients (Travers et al. 2018), a structured tool based on communication skills, workflows and reminder cards (Yu et al. 2017) and audit tools to evaluate the best practices were developed by nurses to be implemented as part of the best practice (Becker et al. 2020; Chong et al. 2013). In one study regarding improving the quality of care for hospitalised patients with cognitive impairment (Travers et al. 2018), nurses developed resources (e.g. card games, camouflage aprons/fiddle blankets) for patients to use whilst in hospital as part of the implemented best practice.

## Discussion

This review highlighted five definite roles nurses play in the implementation of best practices: leadership, collaboration, education and training, communication and feedback and development and tailoring of the best practice. The importance of the leadership role nurses play in this regard was also discussed elsewhere (Bianchi et al. 2018; Vogel et al. 2021). In this review, multiple sub-roles in the nurses’ leadership role in the implementation of best evidence were identified, including recruitment, developing the educational intervention and data analysis. However, it seems from this study that behaviour such as role-modelling, plays a big role in the success of practice change, as found elsewhere (Whitby 2018). Furthermore, for nurses to be equipped for this leadership role, they need to have the necessary educational and managerial support and resources required for implementation of best practices (Bianchi et al. 2018).

Education and training were found to be one of the major roles, with multiple benefits, that the nurse can play in changing practice. These findings confirmed those of Davis and D’Lima (2020), who found that teaching and training initiatives can build capacity in dissemination and implementation of best practices. However, the authors also found a need to increase the number of training opportunities to enhance the number of researchers and practitioners who implement best practices.

Changing practice was often carried out through a collaborative effort with other (specialist) nurses and stakeholders, as part of an interdisciplinary team. The concept of the (interdisciplinary) team approach is widely accepted as the ‘gold standard’ of care delivery globally, influencing patient, nursing and organisational outcomes and policy development which, taken together, are aspired for achievement of high-quality care (Ansell, Sørensen & Torfing 2017; Soukup et al. 2018). Collaboration in changing practice should be fostered through engagement and involvement (Holmes et al. 2019), preferably early in implementation as, from the studies included, collaboration showed multiple benefits. Furthermore, evidence-based practice also includes the patient and families as part of clinical decision-making. However, the nurses’ collaboration with the patient during the implementation of best practices was not highlighted in most included studies. Therefore, the collaborative roles of nurses with patients and families when implementing best practices should be further explored.

The nurse also had a role in ongoing communication and feedback when implementing best practices. Doing so could improve care for an increased number of patients and enhance cost-effectiveness (Brown et al. 2019). Leaders also have a role in enhancing the facilitation of communication. It is important that they are trained in using various platforms for communication in order to facilitate the implementation of the best practice.

Nurses also had a role in development and tailoring of the best practice. As the included studies were conducted in different clinical contexts, with different resources, using a variety of implementation strategies, a needs assessment and intervention mapping – which refers to planning the implementation of best practices based on using theory and evidence – could assist in systematically tailoring a best practice for both nurses and patients and their families (Van Belle et al. 2018).

These identified five roles are interrelated but equally crucial in order to implement best practices. For example, the leadership role will not be fully executed without education and training or collaboration. Communication was found to enhance teamwork (Bayly et al. 2018).

This review found several best practices and implementation strategies. However, studies were found from predominantly middle- and high-income countries. More nurse-led intervention studies describing the role of nurses in the implementation of best practices could therefore be conducted in lower- and middle-income countries where resources are often limited and where the role of nurses is inclined to be more innovative and cost-effective in order to implement these best practices (WHO 2020). Finally, there is a need for nurse-led quality improvement studies to be conducted to produce Level I (e.g. randomised controlled trials) as no such studies were identified.

## Conclusions

The role of nurses in changing practice by implementing best practices is not always well understood. This study found five interrelated, but equally crucial nurse roles in changing practice through the implementation of best practices, namely leadership, education and training, collaboration, communication and feedback and development and tailoring of the best practice. Further exploration on the roles of nurses in changing practices, using randomised controlled trials, including low- and middle-income settings, is required. The study’s findings and identified gaps can be used for further nursing research and education to improve the implementation of best practices and enhance the role nurses can play in this process, thus enhancing patient, nursing and organisational outcomes.
